# Reviews and Book Notices

**Published:** 1880-06

**Authors:** 


### Reviews and Book Notices.

ARTICLE X.—DIPHTHERIA; ITS NATURE AND TREATMENT. By Morell Mackenzie, M.D., London, Senior Physician to the Hospital for Diseases of the Throat and Chest, etc. Lindsay & Blakiston. 1879.

This is a work of one hundred pages, and gives in a concise manner the definition and history, etiology, symptoms, paralysis, diagnosis, pathology, prognosis and treatment of this terrible disease. It also has a chapter on laryngo-tracheal diphtheria, one on nasal and one on secondary diphtheria.

The author believes it to be a disease of antiquity, and that under various names it has existed "many thousand years," and that the exciting cause is a *specific contagion.* He speaks of the manner in which the poison enters the system, and the mode in which the poison is diffused.

Six different forms of the disease are described and the symptoms of the various stages in each fully and carefully considered.

We next find a short chapter on paralyses, which, excepting cardiac syncope, are regarded as the only serious sequelæ to the disease.

The chapter on diagnosis is not as full, perhaps, as could be desired, and the author, in his description of catarrhal diphtheria, presents the picture of what many in our midst call follicular or herpetic tonsillitis. Dr. Mackenzie, however, speaks of his hesitation to attribute the symptoms in this mild form to a disease "the very name of which is heard with consternation."

In the chapter on pathology, we find a description of the exudate in the words of Rindfleisch, and the parasitic theory of Oertel, so fully set forth in Ziemssen's Cyclopædia. The changes also which may and frequently do take place in other tissues and organs, in the course of an attack of diphtheria, are here presented.

About everything known in regard to the treatment of this disease is fully considered under four heads; (1) The recuperative agents; (2) the alleged specifics; (3) the antiseptics; and (4) the expectorants.

Of the three concluding chapters, the one on laryngo-tracheal diphtheria (formerly called croup, as the author remarks), is very valuable. It discusses quite fully the identity or non-identity of the two diseases, and presents some facts which I do not now remember to have seen in any other work. The following quotation evidently represents the author's opinions: "To suppose that there are two kinds of follicular inflammations of the larynx, one in which the cause is the diphtheritic poison, and the other in which the cause is some other undiscovered influence, is totally opposed to all probabilities."

The book, to the busy practitioner, cannot but be a most valuable one. It contains, it appears to the reviewer, nearly every thing of known value in regard to this dreadful disease.

C. W. E.

Article XI.—The Student's Guide to Diseases of the Eye. By Edward Nettleship, F.R.C.S., etc. Philadelphia: Henry C. Lea. 1880. Chicago: Jansen, McClurg & Co.

The author deemed it necessary to apologize for the publication of this book by saying that none of the manuals already before the public "appeared exactly to cover the ground most important for the beginner in clinical ophthalmology."

If this phrase means anything, we must regard it as the author's claim that his book covers more exactly than others the ground "most important for the beginner." To investigate the title to this claim was our object in examining this book.

We have found nothing in the body of the book, represented by its part II, in which this manual materially differs from similar books. This part II is devoted to the diseases of the eye; its chapters are arranged upon the anatomical parts of the eye—as in all ophthalmic text-books—and the context is just as crammed as in other manuals.

The part III is a valuable addition, not found in similar books; it is a clear exposé of the intimate relation of many diseases of the eye to general diseases.

But we are sorry to say the first part of the work falls far below the grade we apply to a guide-book which is said to cover exactly the ground most important for the beginners. We know of nothing which is more important for the clinical student than a thorough familiarity with the correct methods of examining the eye. And a book intended to guide a beginner in his attempts at diagnosticating diseases of the eye, must particularly impress upon his mind the necessity and great value of a methodical examination ; it must teach him to proceed always in a systematic way to be sure that nothing will escape his attention ; and, above all, it should make him thoroughly acquainted with the appearance of the normal eye, so that he can appreciate any deviation from the normal appearance. Our experience in clinical teaching has satisfied us that the great difficulty commonly experienced by medical students in recognizing pathological alterations of the conjunctiva and anterior portion of the eyeball, is the natural or necessary consequence of their imperfect acquaintance with the normal appearance of these structures. The fewest students visiting the eye clinics have ever seen a normal conjunctiva or examined a normal iris carefully enough to tell at once a physiological variation of its appearance from a pathological alteration. All text-books on diseases of the eye—the guide-book before us included—are very particular to make the student thoroughly acquainted with the appearance of the normal fundus, because, without this knowledge, he could not tell the normal from the abnormal any better than a boy can tell the difference between wheat and oats unless he learned it. But the same works are strangely non-committal in regard to the appearance of the normal conjunctiva and other important parts of the eye. This seems to us a particularly serious defect in a book expressly written for the guidance and instruction of the beginner.

But if we find fault with the first part of the book for what it does not contain, we cannot even then approve of the arrangement of its contents. Take the second chapter on "External Examination of the Eye." It begins with *irregularity of the corneal surface*, jumps to the *tension of the eyeball*, *mobility of the eyeball and squint*, *diplopia*, *protrusion of the eye*, and then returns to the anterior portion of the eyeball to examine the *blood-vessels visible on the surface of the eyeball*, the *color of the iris*, the *pupils*, and concludes with tests for *field of vision*, *refraction*, *accommodation*, *color-blindness*, etc. Such ill-arranged, disjointed description of the examination of the eye is a very untrustworthy guide to lead a student through the puzzling labyrinth of symptoms to a clear conception of their meaning; an examination after this pattern is too unmethodical to be recommended to a beginner.

But for this defect, the book would make a very useful little handbook for those medical students who have a natural aversion to the larger, more complete text-books on ophthalmology.

F. C. H.

ARTICLE XII.—HEARING AND HOW TO KEEP IT. By Charles H. Burnett, M.D., etc. Philadelphia: Lindsay & Blakiston; 1879; 16 mo., pp. 152. Chicago: Jansen, McClurg & Co. Price, 50 cents.

Among the many useful treatises in the series of the American Health Primers, this little book, from the able pen of Dr. Burnett, should become one of the most useful and popular. The crying ignorance of most people in regard to the proper care of the ear, calls loudly for the dissemination of the knowledge of hygienic rules written for the people in the plain language of the people. For no organ of equal importance in the whole human body is so flagrantly neglected, so roughly used, and so carelessly exposed as the ear. Any old woman's suggestion for the relief of deafness is readily accepted and acted upon; any drops said to cure earache or toothache, from the harmless sweet-oil to the harmful pain-killer, are put into the external auditory canal. On the other hand, people are amazingly indifferent to the most serious dangers for their audition; they make no attempt to stop a running from the ear of a child; they allow catarrh to impair their hearing many years, instead of having its bad effect arrested right at the beginning. But we must not blame the ignorance of the laity alone for this indifference and carelessness. Too often they are encouraged by general practitioners who do not appreciate the grave nature of these aural diseases, and fail to urge the necessity of early and proper treatment. To these practitioners the perusal of this book will amply repay the sacrifice of the brief hour it takes to read it. It is arranged in three parts; Part I is a brief sketch of the anatomy and physiology of the ear; Part II treats of the chief diseases and injuries of the ear; and Part III contains general hygienic rules for the care of the ear in health and in disease. The following quotation, selected from the chapter on foreign bodies in the ear, may serve as a specimen of the author's style: "It may be said, for the comfort of friends and parents, that no bead, button, 'spit-ball,' bean, grain of wheat or maize, or any similar small object which a child could put into his ear, will do any harm, *if let alone* by all save one entirely conversant with the structure of the ear, and properly supplied with instruments for examination and treatment. If your child, therefore, gets any such foreign matter in its ear, rest assured such object has not gone beyond the reach of surgical skill. The harm in such cases *usually* arises from the unskillful means resorted to, in haste and consternation, for its removal. Let it alone until it can be properly removed." For this golden rule alone the book deserves our hearty recommendation.

F. C. H.

Article XIII.—Ophthalmic Out-patient Practice. By Charles Higgins, F.R.C.S., etc. Second edition. Philadelphia: Lindsay & Blakiston. 1879. Chicago: Jansen, McClurg & Co.

This is a specimen of the "multum in parvo" pattern, touching, in 116 small pages, upon everything, from "a discharge from the eyes" to the ophthalmoscope and the diseases of the fundus. Such rambles are very unprofitable for a student, for he finds—to use an expressive German phrase—*Zu wenig zum Leben und zu viel zum Sterben* (not enough to live and too much to die with hunger). F. C. H.

